# High levels of oxidized fatty acids in HDL impair the antioxidant function of HDL in patients with diabetes

**DOI:** 10.3389/fendo.2022.993193

**Published:** 2022-10-20

**Authors:** Juan Feng, Yunfeng Wang, Weixi Li, Yue Zhao, Yi Liu, Xingang Yao, Shuwen Liu, Ping Yu, Rongsong Li

**Affiliations:** ^1^ College of Health Science and Environmental Engineering, Shenzhen Technology University, Shenzhen Guangdong, China; ^2^ Department of Endocrinology, Shenzhen Sami Medical Center (The Fourth People’s Hospital of Shenzhen), Shenzhen Guangdong, China; ^3^ Clinical Laboratory, Shenzhen Sami Medical Center (The Fourth People’s Hospital of Shenzhen), Shenzhen Guangdong, China; ^4^ National Medical Products Administration Key Laboratory for Research and Evaluation of Drug Metabolism, Guangdong Provincial Key Laboratory of New Drug Screening, School of Pharmaceutical Sciences, Southern Medical University, Guangzhou Guangdong, China

**Keywords:** high-density lipoprotein, diabetes, antioxidant activity, oxidized fatty acids, cardiovascular disease

## Abstract

**Aims:**

Previous studies demonstrate that the antioxidant functions of high-density lipoprotein (HDL) are impaired in diabetic patients. The composition of HDL plays an important role in maintaining the normal functionality of HDL. In this study, we compared the levels of oxidized fatty acids in HDL from diabetic subjects and non-diabetic healthy controls, aiming to investigate the role of oxidized fatty acids in the antioxidant property of HDL.

**Methods:**

HDL was isolated from healthy subjects (n=6) and patients with diabetes (n=6, hemoglobin A1c ≥ 9%, fasting glucose ≥ 7 mmol/L) using a dextran sulfate precipitation method. Cholesterol efflux capacity mediated by HDL was measured on THP-1 derived macrophages. The antioxidant capacity of HDL was evaluated with dichlorofluorescein-based cellular assay in human aortic endothelial cells. Oxidized fatty acids in HDL were determined by liquid chromatography-tandem mass spectrometry. The correlations between the levels of oxidized fatty acids in HDL and the endothelial oxidant index in cells treated with HDLs were analyzed through Pearson’s correlation analyses, and the effects of oxidized fatty acids on the antioxidant function of HDL were verified *in vitro*.

**Results:**

The cholesterol efflux capacity of HDL and the circulating HDL-cholesterol were similar in diabetic patients and healthy controls, whereas the antioxidant capacity of HDL was significantly decreased in diabetic patients. There were higher levels of oxidized fatty acids in HDL isolated from diabetic patients, which were strongly positively correlated with the oxidant index of cells treated with HDLs. The addition of a mixture of oxidized fatty acids significantly disturbed the antioxidant activity of HDL from healthy controls, while the apolipoprotein A-I mimetic peptide D-4F could restore the antioxidant function of HDL from diabetic patients.

**Conclusion:**

HDL from diabetic patients displayed substantially impaired antioxidant activity compared to HDL from healthy subjects, which is highly correlated with the increased oxidized fatty acids levels in HDL.

## Introduction

Epidemiological studies report that patients with diabetes suffer significantly increased cardiovascular disease (CVD) morbidity and mortality. High-density lipoprotein (HDL) in plasma normally functions with antioxidant (1), endothelial-protective (2) anti-inflammatory (3), and anti-atherogenic (4) properties by preventing oxidation of low-density lipoprotein (LDL) and promoting cholesterol efflux from the arterial wall. The antioxidant and anti-inflammatory activities of HDL are impaired in type 2 diabetes compared to healthy control subjects (5, 6). The mechanisms underlying the loss of the beneficial effects of HDL in diabetic patients are likely multifactorial, and compositional changes in the HDL proteome and lipidome may be key players.

HDL is an assembly of a neutral lipid core and an outer shell consisting of polar lipids and proteins. The antioxidant function of HDL is partially mediated by the surface proteins such as apolipoprotein A-I (apoA-I), paroxonase-1 (PON1), and platelet-activating factor-acetyl hydrolase (PAF-AH) (4). In addition, lipidome composition also plays an important role in maintaining HDL function. Lipid peroxidation products of HDL are derived from the degradation of polyunsaturated omega-6 fatty acids, arachidonic acid (AA), and linoleic acid (LA), which are further converted to bioactive eicosanoids through the cyclooxygenase and lipoxygenase pathway, which leads to the formation of hydroxyeicosatetraenoic acids (HETEs) and hydroxyoctadecadienoic acids (HODEs) (7). Previous work shows increases in HODEs and HETEs in the HDL of patients with diabetes and atherosclerotic vascular disease (8), in the plasma of humans and rodents with pulmonary hypertension (9, 10), and in the HDL and LDL from patients with rheumatoid arthritis (11). However, whether the levels of oxidative lipids in HDL from diabetic patients are directly associated with the impaired function of HDL in diabetes still remains elusive. In the present study, we examined the level of oxidized fatty acids in HDL from healthy and diabetic subjects, and as well as whether these changes play a role in the antioxidant function of HDL.

## Materials and methods

### Study population

For Study 1, six consecutive patients with diabetes without known CVDs and other complication syndromes between 18-80 years, as well as six age- and sex-matched healthy control subjects without diabetes were enrolled in this study from the health examination center of Shenzhen Sami International Medical Center within a two-month period. The diagnostic criteria for diabetes in the present study was as follows: a fasting plasma glucose ≥ 7.0 mmol/L and a 2-hour plasma glucose value in a 75 g oral glucose tolerance test ≥ 11.1 mmol/L or a glycated hemoglobin A1c (HbA1c) ≥ 6.5%, as defined by the American Diabetes Association (ADA) (12). The inclusion criteria for diabetes participants was HbA1c ≥ 9% and fasting glucose ≥ 7 mmol/L, and the exclusion criteria was as follows: cardiovascular disease, previous diagnosis of cancer, and moderate-severe chronic kidney or liver disease. Age- and sex- matching in this study refers to that a pair of diabetic subject and healthy control is of the same sex and the age variation is no more than 2 years. Characteristics of participants in Study 1 are shown in **Table 1**. To verify the result of Study 1, another six pairs of diabetic and healthy control subjects with the same inclusion criteria and exclusion criteria were enrolled in Study 2 from the Outpatient Department of Shenzhen Sami International Medical Center within one month. Characteristics of participants in Study 2 are shown in **Table 2**. Venous blood was collected from all study subjects after fasting. Serum was separated by the local clinical laboratory and stored at 4°C for up to 1 week before HDL isolation. The study protocol was approved by the ethics committee of Shenzhen Sami International Medical Center (Ethical Approval No.: SMCC-2022-15).

**Table 1:** Clinical and laboratory characteristics of diabetic patients and non-diabetic healthy controls in Study 1.

	Diabetic (n = 6)	Healthy (n = 6)	*p* value
Age, y	49 ± 10	50 ± 9	0.17
Sex, male/female	4/2	4/2	1.0
BMI, kg/m^2^	24.9 ± 2.4	25.9 ± 4.4	0.73
HbA1c, %	10.9 ± 1.3	5.6 ± 0.4	<0.001
Fasting glucose, mmol/L	12.9 ± 2.2	5.5 ± 0.2	<0.001
TC, mmol/L	5.0 ± 1.2	4.6 ± 1.2	0.23
LDL-c, mmol/L	3.6 ± 1.2	3.2 ± 1.0	0.44
HDL-c, mmol/L	1.0 ± 0.3	1.2 ± 0.2	0.32
TG, mmol/L	2.2 ± 0.8	1.2 ± 0.5	<0.05
ALT, U/L	28.8 ± 15.7	27.0 ± 6.9	0.83
AST, U/L	21.8 ± 8.7	25.5 ± 4.8	0.51

Values are expressed as the mean ± SD or number of subjects. *p* values were obtained from analysis of paired Student’ s t tests. HbA1c, hemoglobin A1c; BMI: body mass index; TC: total cholesterol; LDL-c: low-density lipoprotein cholesterol; HDL-c: high-density lipoprotein cholesterol; TG: triglycerides; ALT: alanine transaminase; AST: aspartate transaminase.

**Table 2:** Clinical and laboratory characteristics of diabetic patients and non-diabetic healthy controls in Study 2.

	Diabetic (n = 6)	Healthy (n = 6)	*p* value
Age, y	51 ± 9	51 ± 9	1.0
Sex, male/female	3/3	3/3	1.0
HbA1c, %	9.3 ± 0.3	5.5 ± 0.3	<0.001
Fasting glucose, mmol/L	10.5 ± 3.0	5.2 ± 0.3	<0.01
TC, mmol/L	5.0 ± 1.6	4.5 ± 1.5	0.64
LDL-c, mmol/L	3.2 ± 0.9	2.8 ± 1.4	0.62
HDL-c, mmol/L	1.1 ± 0.4	1.2 ± 0.3	0.68
TG, mmol/L	2.4 ± 2.5	1.6 ± 0.8	0.58
ALT, U/L	22.2 ± 10.4	19.5 ± 6.3	0.65
AST, U/L	16.3 ± 5.90	16.8 ± 3.2	0.98

Values are expressed as the mean ± SD or number of subjects. *p* values were obtained from analysis of paired Student’ s t tests. HbA1c, hemoglobin A1c; TC, total cholesterol; LDL-c, low-density lipoprotein cholesterol; HDL-c, high-density lipoprotein cholesterol; TG, triglycerides; ALT, alanine transaminase; AST, aspartate transaminase.

### Isolation of HDL

HDL was isolated from the fasting serum of non-diabetic healthy subjects and diabetic patients by dextran sulfate precipitation using a commercially available kit from Cell Biolabs (STA-607, CA), as described previously (13). The purified HDL was subsequently dialyzed in PBS using Slide-A-Lyzer MINI Dialysis devices (10K MWCO, Thermo Fisher, MA). The protein levels of HDL were measured using a bicinchoninic acid (BCA) kit (Thermo Fisher, Waltham, MA) and further identified through sodium dodecyl sulfate polyacrylamide gel electrophoresis (SDS-PAGE). The concentrations of HDL used in the present study were based on protein content of HDL.

### Cell culture

Human aortic endothelial cells (HAECs) were purchased from LMAI Bio (Shanghai, China), and were cultured in endothelial cell medium (ScienCell Research Laboratories, Carlsbad, CA) consisting of basal medium, endothelial cell supplement, 2% fetal bovine serum (FBS) and 1% penicillin/streptomycin antibiotic solution (Hyclone, Logan, UT). THP-1 monocytes were obtained from the National Infrastructure of Cell Line Resource (Beijing, China), and cultured in RPMI 1640 medium (Hyclone) supplemented with 10% FBS and 1% penicillin-streptomycin (Hyclone). All cells were maintained at 37°C in a humidified atmosphere of 5% CO_2_ and were used within 10 passages.

### Measurement of cellular cholesterol efflux capacity

The capacity of HDL to support cholesterol efflux was analyzed in the human monocyte cell line THP-1, a widely-used *in vitro* model for cholesterol efflux (14). N−(7−nitrobenz−2−oxa−1,3−diazol−4−yl)amino)−23,24−bisnor−5−xholen−3β−ol (NBD)−cholesterol was used as an substitute for [^3^H]-cholesterol for the measurement of cholesterol efflux in THP-1 derived macrophages (14). THP-1 cells were seeded in black 96-well plates (Corning Inc., New York, NY) at a density of 1.5^10^4^ cells per well in triplicates and incubated with 100 ng/mL of phorbol-12-myristate-13-acetate (PMA, Sigma-Aldrich, MO) for 72 h. The PMA-differentiated macrophages then were incubated in phenol red-free RPMI1640 medium containing 5 μM NBD−cholesterol (N1148, Thermo fisher) for 4 h at 37°C. Following incubation, the cells were rinsed with PBS three times and were then incubated with HDL (100 μg/mL), as lipid acceptors. The cells were harvested after 4 h, and the medium and cell lysate were collected for the detection of fluorescence intensity. The percentage of NBD-cholesterol efflux was calculated by dividing the fluorescence intensity in the medium by the sum of the fluorescence intensity in the medium and inside the cells.

### Measurement of intracellular reactive oxygen species

The production of ROS was evaluated with an oxidation-sensitive fluorescent probe, 2’,7’-Dichlorodihydrofluorescein-diacetate (DCFH-DA). DCFH-DA is a non-fluorescent lipophilic ester that easily penetrates the cells and could be oxidized into 2’,7’-dichlorofluorescein (DCF) in the presence of oxidants, resulting in a green fluorescence. Therefore, the fluorescence intensity of DCF indicates the levels of ROS in the cells. In brief, HAEC cells were grown in 96-well culture plates with a density of 2^10^4^ cells per well overnight and then pretreated with 10 μM DCFH-DA (Beyotime Biotech Inc., Shanghai, China) in serum-free medium at 37°C for 20 min. Then cells were washed with serum-free medium for three times and further exposed to different treatments for 30 min, and the fluorescence of each well was measured using a multi-plate reader at an excitation wavelength of 485 nm and an emission wavelength of 525 nm. The fluorescence intensity produced by intracellular DCF was used to indicate the oxidant stress in cells.

### Lipidomic analysis of oxidized fatty acids in HDL

A reversed-phase liquid chromatography/tandem mass spectrometry (LC-MS/MS) method was developed to simultaneously analyze oxidized fatty acids including AA and its metabolites such as prostaglandins (PGs), thromboxane (TXB_2_), epoxyeicosatrienoic acids (EETs), dihydroxyeicosatrienoic acids (DHETs), HETEs, and HODEs. To extract the oxidized fatty acids in HDL, 100 μg HDL (indicated by protein content) was mixed with cold methanol (with deuterium labeled 15-HETE-d8 and AA-d8 as internal standards) and formic acid. Then, ethyl acetate extraction was performed for sample preparation after sonication in an ice bath (3 sec on/2 sec off for 60 cycles at 400w), and the organic phase was dried under vacuum and dissolved in methanol for LC/MS-MS analysis. LC/MS-MS was performed using a quadrupole mass spectrometer (Applied Biosystems Division, Life Technologies, Carlsbad, CA) equipped with an electrospray ionization (ESI) source. Chromatography was performed on an Agilent 1290 system (Agilent Technologies Inc., MA, USA) using a Waters symmetry C18 column (250 mm×4.6 mm, 5 μm) with a security guard cartridge maintained at 35°C. The mobile phase was composed of a gradient elution of (A) acetonitrile and (B) water containing 0.05% formic acid (v/v) at a flow rate of 1 mL/min with an injection volume of 10 μL. Metabolites were separated within 50 min with a gradient elution procedure as follows: 0-10 min, 40% A; 10-20 min, 40%-65% A; 20-35 min, 65% A; 35-40 min, 65%-90% A; 40-50 min, 90% A. Mass spectrometric detection was accomplished using multiple reaction monitoring (MRM) mode with negative electrospray ionization.

### Bioinformatics of lipidomic analysis

The analysis workflow of differential metabolites was performed on the ONE-MAP analytical platform (Dashuo Biotech Co., Dalian, China). In brief, the processed peak intensity data acquired from mass spectrometric detection was subjected to multivariate statistical analysis. Linear transformation was used to preserve the variance of the original data in the lower dimensionality of the output data using principal component analysis (PCA) score plots. Significantly differential metabolites were identified using partial least squares-discriminant analysis (PLS-DA). The heatmap was produced in ONE-MAP. In brief, peak intensities of metabolites with a fold change ≥1.5 and *p* value <0.05 were normalized and a heatmap was generated using the pheatmap function. Colors in the heatmap correspond to normalized abundance of each metabolite by a gradient of color from blue (low abundance) to red (high abundance).

### Statistical analysis

The results of all experiments are expressed as the means ± standard deviation (S.D.). All distributions of data were assumed to be normal. Statistical analysis was performed using paired Student’s *t* test with Graphpad Prism Software version 7. The Pearson’s correlation coefficient assumes that X and Y are jointly distributed as bivariate normal, i.e., X and Y each are normally distributed, and that they are linearly related (15). Pearson’s correlation analysis between variables were performed in an all-group comparison method when applicable (16). A *p* value < 0.05 was considered statistically significant.

## Results

### Demographic, laboratory, and clinical characteristics of the Study 1 population

The characteristics of the two groups in Study 1 are shown in **Table 1**. The gender and age distribution, as well as the body mass index (BMI) were similar between the two groups. Glycemic control was poor in diabetic patients (HbA1c: 10.9 ± 1.3%). The serum levels of fasting glucose and HbA1c in the diabetic group were markedly higher than that in the healthy control group (HbA1c: 10.9 ± 1.3% vs 5.6 ± 0.4%, *p*<0.001; fasting glucose: 12.9 ± 2.2 mmol/L vs 5.5 ± 0.2 mmol/L, *p*<0.001). There was no significant difference in the serum levels of total cholesterol (TC), LDL-cholesterol (LDL-c), HDL-cholesterol (HDL-c), alanine transaminase (ALT), or aspartate transaminase (AST) between the diabetic and healthy group, except that serum triglycerides (TG) was slightly higher in diabetic patients compared with healthy controls (2.2 ± 0.8 mmol/L vs 1.2 ± 0.5mmol/L, *p*<0.05) (**Table 1**).

### HDL from diabetic patients is pro-oxidative compared to HDL from healthy control subjects

**Figure 1:**
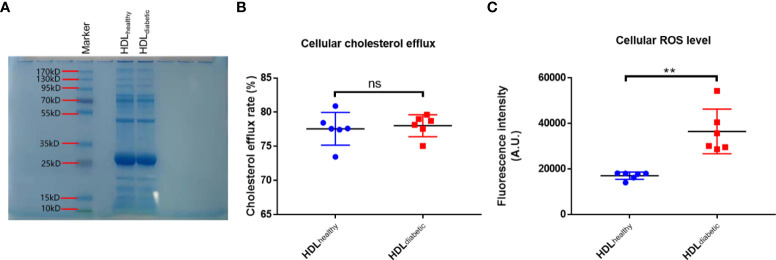
Analysis of the pro-oxidative activity of HDLs from diabetic patients. **(A)** SDS-PAGE analysis of HDLs purified by the sulfate dextran precipitation method. Ten micrograms of HDL purified using a commercial kit was loaded on a 4-20% Bis-Tris gel and stained with Coomassie Brilliant Blue dye. **(B)** Comparison of cholesterol efflux capacity of HDL from diabetic patients and healthy controls. Differentiated THP-1 cells were loaded with NBD-cholesterol for 4 h, then treated with 100 ug/mL HDL from healthy controls (n=6) or diabetic subjects (n=6) for another 4 h. The fluorescence intensity in cells and in the medium was determined. Cholesterol efflux rate was calculated by dividing the fluorescence intensity in the medium by the sum of fluorescence intensity in the medium and cells. **(C)** Comparison of the level of ROS in HAECs treated with HDL from healthy subjects and diabetic patients. HAECs were pre-incubated with 10 uM DCFH-DA for 20 min, then treated with 500 ug/mL HDL from healthy controls (n=6) or diabetic subjects (n=6) for 30 min. The intracellular fluorescence intensity produced by DCF was determined. ns: not significant. **p < 0.01, ns: not significant. *p* value was obtained from analysis of paired Student’ s *t*-tests.

HDLs were isolated from the serum of the subjects in the two groups in Study 1. SDS-PAGE analysis of HDLs is shown in **Figure 1A**, displaying a main band comparable to apoA-I (the major apolipoprotein in HDL, molecular weight: 28 kD). The cholesterol efflux capacity and the effect on ROS production of HDLs were examined on THP-1 and HAEC cells, respectively. THP-1 derived macrophages were loaded with NBD-cholesterol firstly, and then HDL was added to induce the efflux of NBD-cholesterol. It was found that there was no difference in the ability of HDL to induce cholesterol efflux between diabetic patients and controls (**Figure 1B**). To compare the effect of HDL from the two groups on the oxidation status of cells, HAEC cells were treated with HDLs from diabetic patients (HDL_diabetic_) and healthy controls (HDL_healthy_), respectively, and the levels of intracellular ROS, an oxidant index was analyzed as indicated with the fluorescence intensity of DCF. As shown in **Figure 1C**, the ROS levels in cells treated with HDL_diabetic_ was significantly higher than that in cells treated with HDL_healthy_, indicating that HDL_diabetic_ was pro-oxidant compared to HDL_healthy._

### Oxidized fatty acids were increased in HDL from patients with diabetes compared to healthy controls

**Figure 2:**
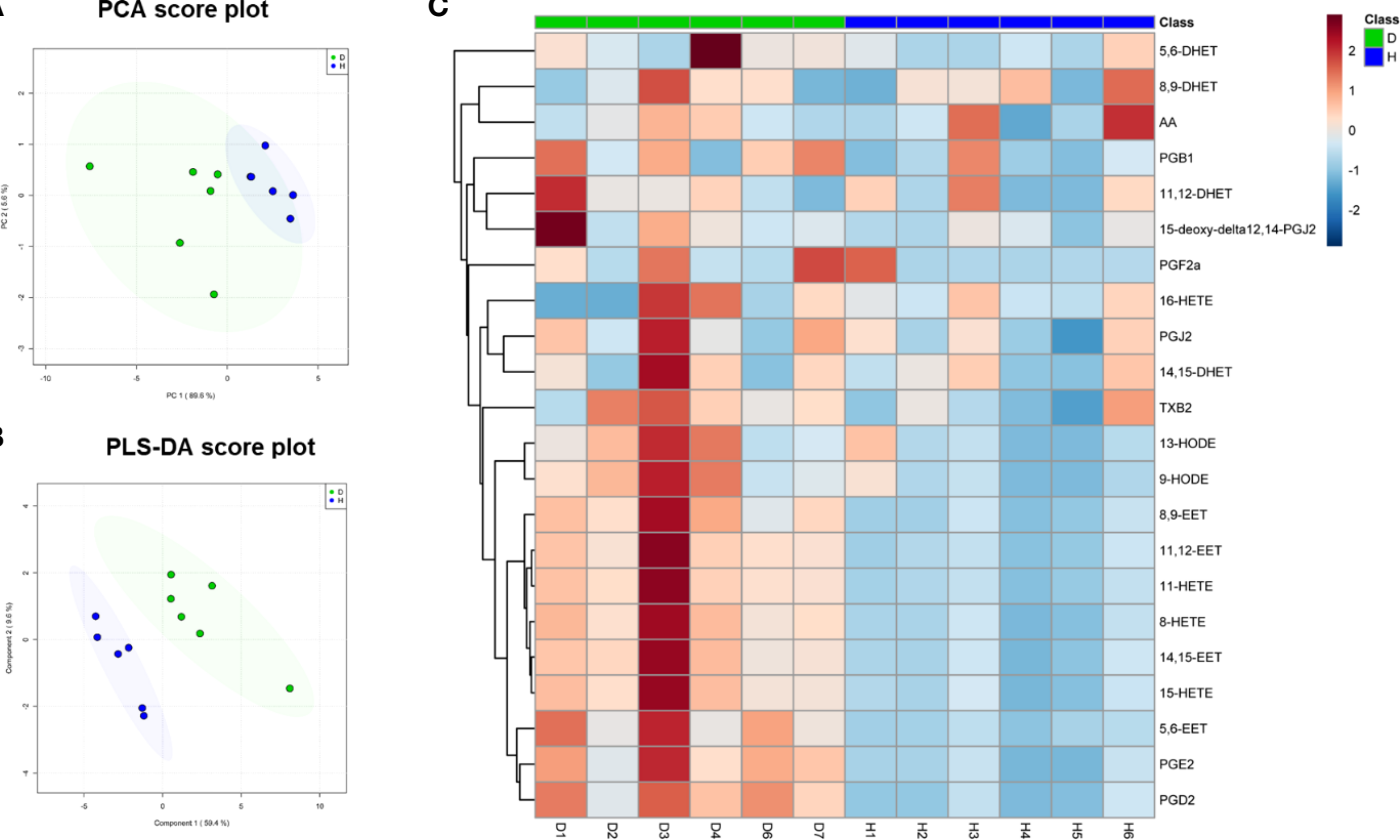
Multivariate analysis of the oxidized fatty acid lipidome of HDL from diabetic subjects and healthy controls in Study 1. **(A)** Principal Component Analysis (PCA) and **(B)** Partial Least Squares Discriminant Analysis (PLS-DA) of HDL from diabetic patients (D, n=6) and healthy subjects (H, n=6). The shaded areas indicate 95% confidence ellipse regions based on the data points for individual groups. **(C)** Heatmap visualization based on the significantly changed oxidized fatty acids (fold change ≥1.5, *p*<0.05). Colors in the heatmap correspond to normalized abundance of each metabolite by a gradient of color from blue (low abundance) to red (high abundance).

**Figure 3:**
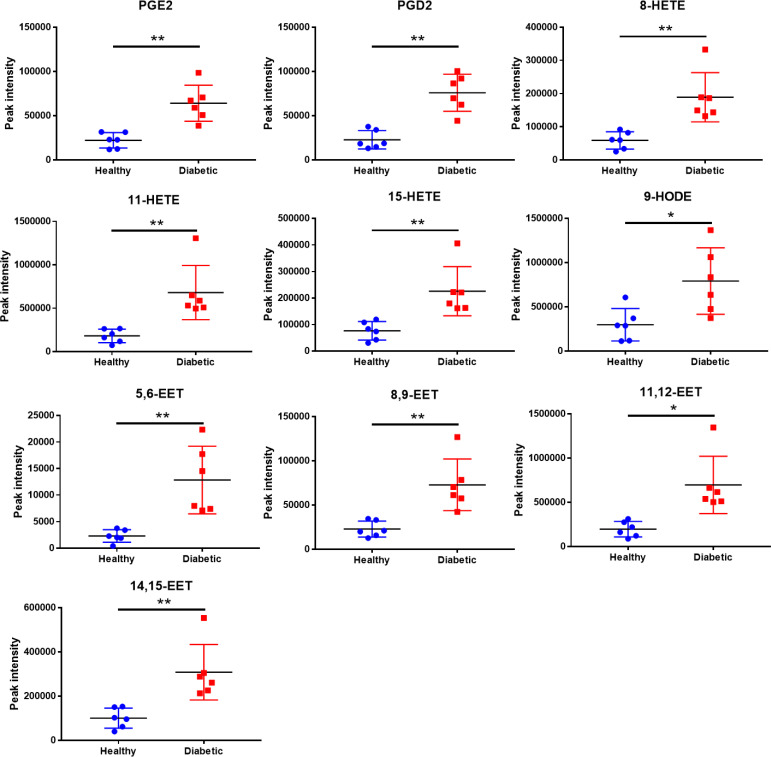
Quantitative comparison of oxidized fatty acids that significantly changed between the diabetic and healthy groups. **p < 0.01, *p < 0.05. *p* values were obtained from analysis of paired Student’ s *t*-tests.

The levels of oxidized fatty acids in HDLs from subjects in Study 1 were measured by LC/MS-MS. To compare the overall features between the two groups, PCA was performed to visualize the global variations between the two groups. PCA of all detected features unveiled two distinct cluster marked by HDLs from diabetic and healthy subjects, respectively (**Figure 2A**). In particular, the diabetic group clustered in the negative sector of PC1, while the healthy group clustered in the positive sector of PC1. The PCA score plot revealed a good separation especially along the first principal component PC1 (PC1 and PC2 accounted for 89.6% and 5.6% of the total variance, respectively). This result indicates that differences were present between the two groups. Then, supervised classification using PLS-DA model was also performed to search for discriminating features for the separation between the two groups. A clear separation between the two groups was also observed in the PLS-DA score plot (**Figure 2B**), confirming the distinct features between the two groups. Moreover, a heat map of all of the differential metabolites was produced to visualize the relative concentration of oxidized fatty acids in each sample. The relative concentration of each metabolite in each sample is expressed with different colors. It is clear that there were higher levels of oxidized fatty acids in HDL from diabetic patients compared to the healthy control group (**Figure 2C**). Among the 19 studied metabolites, 10 oxidized fatty acids with a false discovery rate (FDR) <0.05, fold-change >2 were further picked out. As can be seen in **Figure 3**, the levels of the ten oxidized metabolites of AA and LA, including PGs, HODE, HETEs, and EETs, were significantly elevated in HDL from patients with diabetes.

### The levels of oxidized fatty acids in HDL were positively correlated with the oxidant index of HDL-treated cells

**Figure 4:**
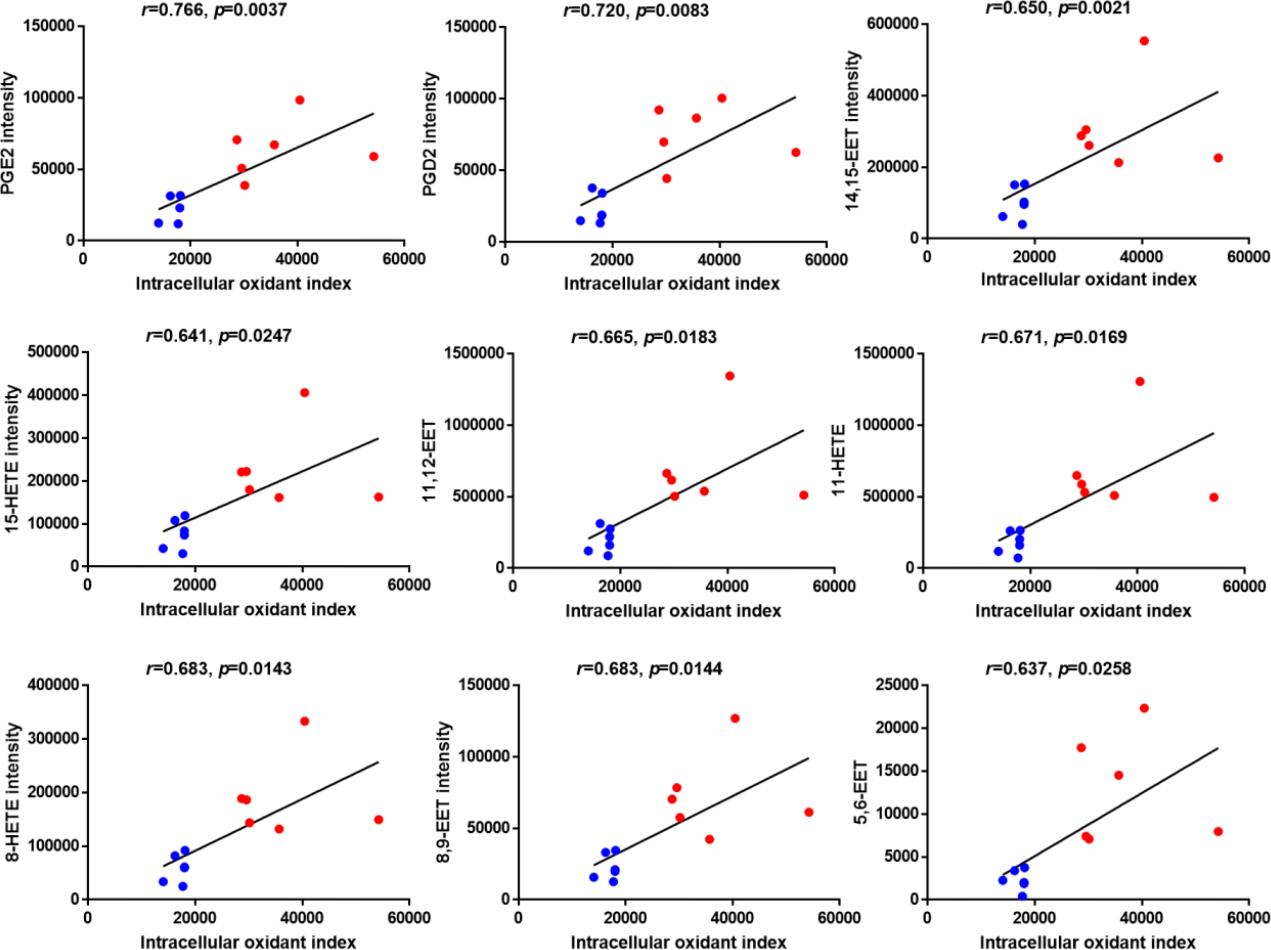
Correlation analysis between the levels of significantly changed oxidized fatty acids and the intracellular oxidant index of HAECs treated with HDLs determined by Pearson’s analysis. Blue dots indicate HDL from healthy control group and red dots indicate HDL from diabetic group. N=6 for each group.

To investigate the correlation between elevated oxidized fatty acids and impaired HDL function, Pearson’s correlation analyses were performed between the oxidant index of HAEC cells treated with each HDL sample as shown in **Figure 1C** and the intensity of the 10 significantly changed oxidized fatty acids as shown in **Figure 3** after confirming that the assumptions of Pearson’s correlation were satisfied. Despite the small numbers in the subset of subjects, Pearson’s correlation analyses demonstrated strong positive correlations between the levels of 9 significantly changed oxidized fatty acids except 9-HODE (data not shown) and the cellular oxidant index with *r* values > 0.6 and *p* values < 0.05 (**Figure 4**).

### Elevated levels of oxidized fatty acids lead to impaired antioxidant capacity of HDL

**Figure 5:**
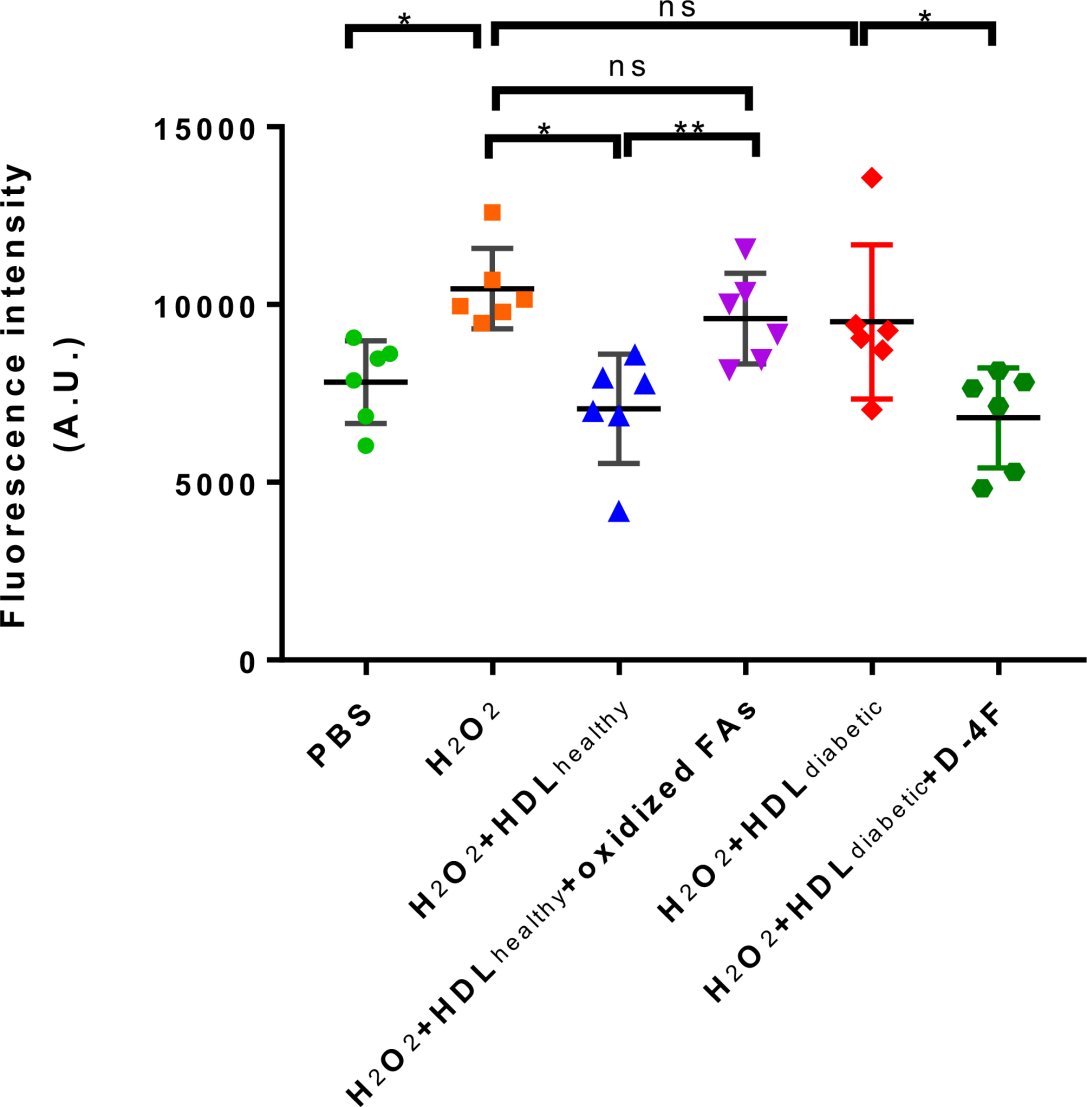
The effects of oxidized fatty acids and D-4F on the antioxidant functions of HDLs. HAECs were pre-incubated with 10 uM DCFH-DA for 20 min, then treated with PBS, 500 uM H_2_O_2_, H_2_O_2_ plus 500 ug/mL HDL from healthy controls (n=6), H_2_O_2_ plus 500 ug/mL HDL from healthy controls (n=6), and a mixture of oxidized fatty acids (FAs) composed of 20 ng/mL PGD2, 9-HODE, 8-HETE, and 5(6)-EET, H_2_O_2_ plus 500 ug/mL HDL from diabetic subjects (n=6) and 50 ug/mL D-4F for 30 min, respectively. The intracellular fluorescence intensity produced by DCF was determined. **p < 0.01, *p < 0.05, ns: not significant. *p* values were obtained from analysis of paired Student’ s *t*-tests.

Oxidative stress results in excessive accumulation of ROS, which directly damages cell membranes, protein and DNA, compromising cell function and threatening cell survival (17, 18). Other researchers have shown that the effects of experimentally induced ROS induced in the vascular wall were completely abolished by a daily infusion of HDL (1). HDL also possess the ability to protect endothelial cells from primary apoptosis and to reduce intracellular ROS induced by oxidized LDL (19). In addition, HDL has also been shown to protect mesenchymal stem cells from hydrogen peroxide (H_2_O_2_)-induced oxidative stress and apoptosis (20). The pro-oxidative property of diabetic HDL has been revealed in **Figure 1C**. To further investigate whether the impaired antioxidant function of HDL from diabetic patients is caused by elevated levels of oxidized fatty acids, *in vitro* DCF-based cell assays were performed with individual HDLs from subjects of Study 2 (characteristics of the study population see **Table 2**). H_2_O_2_, which is widely used as an oxidant, was applied to induce an oxidative stress model in HAEC cells. As shown in **Figure 5**, H_2_O_2_ caused an evident increase in cellular ROS, while HDLs isolated from healthy subjects substantially reduced H_2_O_2_-stimulated oxidative stress, indicating a potent antioxidant property of HDL from healthy subjects on the endothelium. However, the addition of 20 ng/mL a mixture of oxidized fatty acids (PGD_2_, 9-HODE, 8-HETE, and 5(6)-EET) significantly disrupted the antioxidant function of HDL_healthy_ (**Figure 5**). Compared to HDL from healthy controls, HDL from diabetic patients lost the capacity to protect endothelial cells from oxidative stress induced by H_2_O_2_ (**Figure 5**). The apoA-I mimetic peptide 4F, which forms a class A amphipathic helix similar to those found in apoA-I, is reported to have anti-inflammatory and antioxidant effects due to its binding to oxidized fatty acids such as HETEs and HODEs (21), and improve the function of HDL (22). To determine whether 4F could restore the impaired antioxidant function of HDL, HAEC cells were co-incubated with D-4F and dysfunctional HDL_diabetic_. Incubation of HDL with 50 μg/mL D-4F rescued the antioxidant capacity of HDL_diabetic_ (**Figure 5**). These results strongly indicate that the reduced antioxidant capacity of HDL is, at least partially, caused by increased oxidized fatty acids.

## Discussion

CVD remains the leading cause of death in the developed and developing countries, and is prevalent in diabetes mellitus with 68% CVD-related mortality (1, 17). Circulating HDL plays cardiovascular protective roles by promoting cholesterol efflux and antioxidant effects, *etc* (2–5). In the present study, we disclosed that there was a dramatic descend in the antioxidant capacity of HDL from diabetic patients while the cholesterol efflux capacity was not altered. There was also an abnormal increase of oxidized fatty acids inside HDL, which was associated with the impaired antioxidant activity of HDL in diabetes.

Previous epidemiological studies demonstrate strong, inverse associations between HDL-c and CVD (23). However, efforts to raise HDL-c level *via* CETP inhibitors turned out be disappointing in outcome studies despite substantial increases in HDL-c (24, 25), questioning the causal role of HDL-c in CVD risk development (26). Furthermore, researchers proposed that the functional properties, rather than the cholesterol in HDL, are more important indices for HDL-targeted therapies (27). It is in fact the capacity to transport cholesterol from the periphery to the liver, anti-inflammatory properties, antioxidant effects and the endothelial-vasoprotective effects of HDL that renders it atheroprotective (27). Dysfunctional HDL has shown to have strong and independent associations with CVD risk (3, 4, 28) and impaired HDL function in diabetes has been proposed as a mechanism underlying the increased CVD risk in diabetes (2).

Cholesterol efflux capacity is a key metric of the anti-atherosclerotic functionality of HDL. Thus far, variable changes in the cholesterol efflux capacities of HDL from diabetes patients have been reported. While some found decreased (29–33), others reported unchanged (34–36), or even increased HDL cholesterol efflux in diabetic patients (37). The differences in protocols, for example, the cell type (macrophages or fibroblast), the stimulation or not of cholesterol transporters, the tracers (radioactive or fluorescent), the type of acceptor (HDL, total plasma, or apolipoprotein B (apoB)-depleted plasma) are likely to explain the divergent results. It is important to point out that no gold standard is currently agreed upon for cholesterol efflux assays. ATP-binding cassette transporter A1 (ABCA1)-mediated cholesterol efflux is the most important determinant of total efflux from THP-1 macrophages (38). The use of HDL isolated by ultracentrifugation or dextran sulfate precipitation instead of apoB-depleted plasma may also be a source of discrepancy because of the elimination of some pre-β-HDL, which plays an important role in ABCA1-dependent cholesterol efflux (39). In our study, there was no difference in the cholesterol efflux from THP-1 monocytes-derived macrophages either from normal or from diabetic patients. One of the possible reasons might be the concentration of HDL used in the cholesterol efflux assay was relatively low (100 μg/mL), which was lower than that we used in ROS assay (500 μg/mL) and the commonly used concentration of HDL described in other studies (150~800 μg/mL) (34, 40). Another reason might be the lack of pre-β-HDL in the HDLs isolated by the dextran sulfate precipitation which is dominated by mature HDLs (41). Wijtske et al., working with THP-1 macrophages and apoB-depleted plasma, also reported that HDL cholesterol efflux function was not impaired in diabetes but was lower in metabolic syndrome, partly dependent on plasma HDL-c levels (35). Damien et al. also reported that the HDL cholesterol efflux was not altered in type 2 diabetes despite lipidomic abnormalities (34). The surprisingly increased HDL cholesterol efflux in diabetes reported by Low et al. was linked to the higher levels of CETP activity observed in diabetic patients (37), Overall, there is no consensus on how diabetes affects the cholesterol efflux capacity of HDL, and we can conclude that not all of HDL functions are modified with HDL oxidation. As studied here, only the antioxidant function of HDL was impaired by oxidation and can be restored by D-4F, while the capacity of cholesterol efflux remained unchanged.

Although there was no significant difference in HDL-c and the ability to support cholesterol efflux between the two groups, the antioxidant capacity of HDL was significantly dampened in diabetes, confirming that HDL particles can become dysfunctional independent of HDL-c levels. The antioxidative property of HDL is associated with compositional changes in the HDL proteome and lipidome. Proteins compose 35-65% of the molecular weight of HDL particles (42). Thus far, there have been more than 90 protein species identified in HDL. The most commonly observed major protein groups are apolipoproteins, enzymes, lipid transfer proteins, and acute-phase-response proteins. The levels of apolipoproteins and enzymes (43) the posttranslational modification of apolipoproteins (44), and the protein-protein interactions in HDL (45) could affect the functions of HDL.

Beyond the proteome, oxidized lipids including HETEs and HODEs in HDL are also reported to be associated with the higher risk of CVD in populations with diabetes (8). As the oxidized lipid metabolites of AA and LA, HETEs and HODEs have been implicated as important mediators of the immune response (46) and directly implicated in the pathogenesis of atherosclerosis (47, 48). The presence of oxidized fatty acids such as HETEs and HODEs in HDL indicates the occurrence of lipid peroxidation in response to inflammation and oxidative stress. Also, it is possible that oxidation of lipoproteins during the HDL isolation may affect HDL lipid composition. However, it should be noted that HDL from all samples in the present study was isolated under identical conditions using the same methods. Therefore, differences in oxidized fatty acids among groups are less likely due to oxidation of lipoproteins during isolation. Previous studies suggest that hyperglycemia may promote pathological effects through glycation of lipoproteins (49), which leads to increased susceptibility to oxidation (50). This increased oxidative stress contributes to the formation of certain oxidized lipids (47). Therefore, it is plausible that the increased levels of oxidized lipid metabolites in HDL from patients with diabetes is caused by hyperglycemia.

In the current study, the levels of oxidized fatty acids were significantly elevated in the HDL from diabetic patients compared to matched healthy controls. Moreover, high levels of oxidized fatty acids in HDL were significantly associated with worse antioxidant function of HDL. Our study suggests a potential mechanism in which hyperglycemia in diabetic patients results in increased oxidized fatty acids in HDL, thereby leading to impairment in HDL functionality, and increased CVD risk. The main findings of our study are consistent with those by Morgantini et al. (51) that the function of HDL is highly correlated with oxidized fatty acids inside the lipoprotein. There are some variations in the species of oxidized fatty acids detected between two studies, possibly due to differences in the methods for lipoprotein isolation and lipodomic analyses by LC-MS/MS. Besides common HETEs and HODEs, our study also reported the elevations of PGD_2_, PGE_2,_ and EETs that are produced in different ways from HODEs and HETEs in the HDLs of diabetic patients and their correlations with HDL dysfunction, providing additional evidence for the mechanism underlying oxidized fatty acid-leading HDL dysfunction.

ApoA-I, the major protein in HDL is a selective target for oxidation by myeloperoxidase, which results in impaired HDL function. ApoA-1 mimetic peptides may have the ability to modify the lipid and protein content of HDL and convert dysfunctional HDL to functional. The apoA-I mimetic peptide 4F was designed to contain a class A amphipathic helix with a polar and a nonpolar face that allows it to bind lipids (52). Actually, the mimetic peptide 4F bound oxidized fatty acids derived from AA and LA with an astoundingly higher affinity than apoA-1 (53). Oral administration or injection of 4F restore the anti-inflammatory and antioxidant activities of HDL as well as the capacity to promote cholesterol efflux in various models. For example, D-4F could restore the anti-inflammatory properties of HDL after influenza infection in mice by preventing macrophage infiltration into the aortic arch (54). Administration of 13(*S*)-HPODE, a kind of oxidized products of polyunsaturated fatty acids, increased plasma 13-HODE and 9-HODE levels and decreased HDL anti-inflammatory properties in mice, while injection of 4F decreased the levels of 13-HODE and 9-HODE, and remarkably rescued the anti-inflammatory property of HDL impaired by 13(*S*)-HPODE (22). Watanabe et al. reported that administration of 4F rescued the anti-inflammatory activity of HDL by disturbing the association of hemoglobin with HDL in hyperlipidemic mice (55). Meanwhile, oral administration or injection of 4F can also restore the antioxidant activities of HDL as well as the capacity to promote cholesterol efflux in various models. A study by van Lenten et al. found that treatment of mice with D-4F resulted in an increase in HDL-c and PON1 activity, and the ability to inhibit LDL-induced monocyte chemotactic activity (54). Novab et al. reported that oral administration of D-4F led to a substantial increase in the protective capacity of HDL to protect LDL against oxidation and dramatically reduced atherosclerosis in LDL receptor-null mice but independent of changes in total plasma or HDL-c (56). Further mechanism study by the same group revealed that D-4F caused the formation of pre-β-HDL, reduced lipid hydroperoxides in HDL and increased PON1 activity of HDL in mice (57) and monkeys (58). Pre-β-HDL is generally considered to be the most active HDL fraction in promoting reverse cholesterol transport, and the cycling of cholesterol through pre-β HDL is generally considered to be protective against atherosclerosis (59).

How could D-4F decrease HDL lipid hydroperoxide content and increase PON1 activity? PON1 is a HDL-associated enzyme and has the ability to prevent the formation of proinflammatory oxidized phospholipids (60), thereby mainly responsible for the antioxidative properties of HDL. Forte et al. have shown that the activity of PON1 are reversibly inhibited by lipid hydroperoxides (61). It can be concluded that effective sequestration of lipid hydroperoxides by peptides such as D-4F may active PON1, while the active PON1 further reduces the levels of oxidized lipids, finally forming a positive feedback loop (62). Consistent with the fact that administration of the 4F peptide improved HDL function in various *in vivo*, *in vitro* or *ex-vivo* models, herein our results revealed that addition of D-4F reverted pro-oxidant HDL_diabetic_ into antioxidant, supporting the notion that oxidized lipids in HDL are strongly related to HDL function since the effect of the apoA-I mimetic is attributable to its ability to bind oxidized lipids.

Although interesting, there are some limitations to the present study. One limitation is that the findings are kind of hypothesis-driven based on existing literature, and limited by the small sample size, which makes the Pearson’s correlation between oxidized fatty acids and HDL antioxidant functionality based on the two separate clusters should be interpreted prudently. The correlations may not exist when calculated separately in diabetic subjects and in healthy controls, since the range of observed values in the predictor variable was too small. Another limit is the unknown size distribution of the HDL particles due to limited amount of serum samples. HDL particle sizes are differentially associated with and may mediate atherosclerotic CVDs (63). Future studies will evaluate levels of oxidized fatty acids in HDL with different sizes from larger diabetes cohorts in order to better understand how HDL is altered in diabetes and to determine the effect of specific treatments. In addition, further work is warranted to determine whether these assessments of HDL composition beyond HDL-c levels are more useful markers for cardiovascular risk in diabetes patients.

## Conclusions

In summary, the present study indicated the relevance between increased oxidized fatty acids in HDL and the impaired antioxidant function of HDL from diabetic patients, supporting the link between higher CVD risk in patients with diabetes and HDL composition beyond HDL-c concentration.

## Data availability statement

The raw data supporting the conclusions of this article will be made available by the authors, without undue reservation.

## Ethics statement

The studies involving human participants were reviewed and approved by the ethics committee of Shenzhen Sami International Medical Center (Ethical Approval No.: SMCC-2022-15). The patients/participants provided their written informed consent to participate in this study.

## Author contributions

RL and PY conceived and designed the study. JF contributed to conduction of study, data analysis and writing the manuscript. YW, YZ and YL contributed to subject recruitment and data collection. WL contributed to conduction of study. XY and SL contributed the review of the manuscript. All authors contributed to the article and approved the submitted version.

## Funding

The work was supported by the Shenzhen Natural Science Foundation (Grant No. JCYJ20190813141001745), Natural Science Foundation of Top Talent of SZTU (Grant No. 2020102), Shenzhen Science and Technology Program (Grant No. RCBS20210609103650047), Open Foundation of Guangdong Provincial Key Laboratory of New Drug Screening (Grant No. GDKLNDS-2021OF003), Startup Research Fund of Shenzhen Technology University, and the Shenzhen Pengcheng Scholar Program.

## Conflict of interest

The authors declare that the research was conducted in the absence of any commercial or financial relationships that could be construed as a potential conflict of interest.

## Publisher’s note

All claims expressed in this article are solely those of the authors and do not necessarily represent those of their affiliated organizations, or those of the publisher, the editors and the reviewers. Any product that may be evaluated in this article, or claim that may be made by its manufacturer, is not guaranteed or endorsed by the publisher.
